# RPINet: Dual-branch attention-guided fusion of remote sensing images and point clouds for urban scene segmentation

**DOI:** 10.1371/journal.pone.0349557

**Published:** 2026-05-22

**Authors:** Zhe Jing, Zhengguo Yan

**Affiliations:** School of Electronic Engineering, Xi’an Shiyou University, Xi’an‌‌, China; University of Alicante: Universitat d’Alacant, SPAIN

## Abstract

Semantic segmentation of large-scale urban point clouds is a fundamental yet challenging task due to the complex spatial structures, massive data volume, and irregular distribution of points. Existing methods typically rely solely on 3D geometry or naively fuse 2D and 3D features, leading to limited performance in capturing both fine-grained details and global semantic consistency. In this paper, we propose RPINet, a novel **Remote-Projection and Intelligent Network** that effectively integrates **2D remote sensing images** with **3D point cloud data** for enhanced semantic segmentation. RPINet adopts a dual-branch architecture where the 3D point branch extracts spatial features using PointNet++, transformers, and graph convolutional networks, while the 2D image branch leverages a Gaussian splatting projection and CNN-based encoding to retain texture and contour information. A **hybrid attention-based fusion module** dynamically weights intra-modal and inter-modal dependencies, enabling deep semantic interaction between modalities. In addition, we introduce an adaptive sampling strategy and a multi-objective loss function to optimize segmentation accuracy and geometric consistency. Extensive experiments on the challenging SensatUrban dataset demonstrate that RPINet achieves state-of-the-art performance with a mean IoU of 66.5%, outperforming existing methods by a significant margin. Our model also shows strong generalization ability on unseen datasets, confirming its robustness and practical applicability to real-world urban scenarios.

## 1 Introduction

Urban-level point cloud segmentation task is an important basis for realizing semantic scene [1]. Urban-scale 3D point cloud semantic segmentation is fundamental for accurate scene understanding in applications such as autonomous driving, urban planning, and remote sensing. Unlike small-scale indoor environments, urban-scale point clouds exhibit extreme complexity, with billions of points and significant variation in density, occlusion, and noise [2–5]. The challenges in urban-level segmentation are twofold: (1) the irregular and sparse nature of point clouds limits the direct application of standard convolutional methods, and (2) the need to preserve fine-grained geometric features while capturing large-scale spatial context requires models to balance local detail extraction with global reasoning [6–10].

Despite the growing attention on urban-scale semantic segmentation, existing methods still face critical limitations. Traditional point-based approaches, such as PointNet++ and RandLA-Net, are effective in local geometry encoding but lack global contextual reasoning. Voxel-based and sparse convolutional methods suffer from discretization errors and high memory costs. Furthermore, multi-modal fusion techniques that combine 2D imagery with 3D point clouds often struggle with projection-induced information loss and insufficient cross-modal alignment. Recent efforts using cross-attention mechanisms have shown promise but are typically constrained to small-scale indoor settings or well-aligned RGB-D data. Thus, developing an effective and scalable fusion strategy that can fully leverage the complementary strengths of 2D and 3D modalities remains a significant open problem.

To address these challenges, remote sensing data, such as aerial imagery, is often used to complement 3D point clouds with rich texture and color information [11]. However, effectively integrating multi-modal data for robust urban scene segmentation remains a critical problem, especially when projecting 3D geometry into 2D space may cause loss of structural information [12–14].

**Fig 1:**
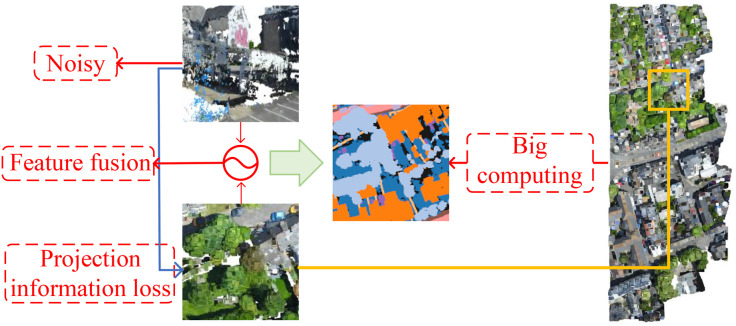
Motivation. From the perspective of remote sensing, using point cloud data for target detection faces numerous challenges that need to be urgently addressed, such as noise interference, feature fusion, information loss, and large computational load. Example image and point-cloud views in this figure are adapted from the SensatUrban dataset by Hu et al. [1] and used under the MIT License from the official SensatUrban repository.

In this paper, we propose RPINet, a Remote-Projection and Intelligent Network that fully leverages the complementary strengths of 2D remote imagery and 3D point clouds for urban-scale semantic segmentation. RPINet introduces a two-branch architecture: the Remote Image (RI) branch performs adaptive denoising and feature enhancement on projected 2D data, while the Point Cloud (PI) branch applies advanced clustering, multi-scale feature extraction, and adaptive sampling directly on the 3D points. The extracted features are fused via a hybrid attention mechanism designed to capture both intra-modal and inter-modal dependencies. Furthermore, we introduce a comprehensive loss function that jointly optimizes information‌‌ preservation during projection and segmentation accuracy [15–17] (Fig 1).

The contributions of this work are summarized as follows:

- We design an adaptive projection method using Gaussian splatting to minimize information loss when converting 3D point clouds into 2D representations.
- We propose an advanced point cloud feature extraction pipeline combining DBSCAN-based clustering, PointNet++, transformer-based attention, and graph convolutional networks (GCN) to enhance multi-scale geometric feature learning.
- We develop a hybrid attention-based cross-modal feature fusion strategy that effectively integrates 2D texture and 3D structural features.
- We introduce a multi-term loss function that balances information preservation, segmentation accuracy, and spatial consistency.

Experimental results on the SensatUrban dataset demonstrate that RPINet achieves superior performance compared to state-of-the-art methods, particularly in preserving fine-grained details and maintaining global semantic coherence across large urban scenes.

The remainder of this paper is organized as follows. Section 2 reviews related work on urban point cloud segmentation and multi-modal fusion. Section 3 introduces the proposed RPINet architecture in detail, including the dual-branch design, fusion mechanism, and loss functions. Section 4 presents experimental evaluations on the SensatUrban dataset, including performance comparisons and ablation studies. Finally, Section 5 concludes the paper and discusses future research directions.

## 2 Related work

### 2.1 Urban-scale point cloud semantic segmentation

With the increasing adoption of LiDAR and UAV technologies, large-scale outdoor point cloud semantic segmentation has become an essential task in urban scene understanding. Unlike indoor scenes, urban-scale point clouds are characterized by vast spatial extents, irregular density, and complex object distributions, which present significant challenges for accurate segmentation. Early approaches relied heavily on handcrafted geometric features, such as curvature, planarity, or height histograms, combined with traditional machine learning classifiers (e.g., random forests, support vector machines). However, these methods often failed to generalize across diverse urban environments due to the limitations of manually designed descriptors.

Voxelization-based methods attempted to discretize point clouds into regular grids for convolutional processing. However, such approaches suffer from high memory consumption and loss of fine geometric details due to quantization errors, especially when scaling to large outdoor scenes.

To address these challenges, deep learning-based methods have emerged, with PointNet++ [18] and RandLA-Net [19] being representative. PointNet++ introduced hierarchical feature learning via local neighborhood set abstraction, capturing fine-grained geometric structures. RandLA-Net proposed random sampling with local feature aggregation to achieve efficient large-scale point cloud processing. Despite these advances, these methods still struggle to capture long-range dependencies and global contextual relationships essential for understanding complex urban environments.

Recent work has explored multi-scale feature extraction and contextual modeling to enhance segmentation. For instance, KPConv [20] uses kernel point convolutions to process irregular neighborhoods, and SPVNAS [21] applies sparse convolution with network architecture search. Nevertheless, the difficulty of effectively balancing local detail preservation with global semantic understanding remains a core limitation in large-scale outdoor point cloud segmentation.

### 2.2 Multi-modal fusion for point cloud segmentation

Given the complementary nature of different data modalities, fusing point clouds with additional information such as RGB images, aerial remote sensing data, or depth maps has proven to enhance semantic segmentation performance. Remote sensing images provide rich texture and color information, while point clouds contribute accurate 3D geometric structure, making their fusion highly desirable for comprehensive scene understanding.

Early fusion strategies projected point clouds onto 2D grids or panoramic images and applied conventional convolutional neural networks (CNNs) to extract features [22]. However, such projections inevitably introduce information loss, as continuous 3D structures are collapsed into discrete 2D spaces, disrupting spatial relationships.

Mid-level and late fusion methods attempt to independently extract features from both modalities and combine them at various network stages. Techniques such as feature concatenation, addition, and multi-branch architectures have been widely adopted [23]. While these approaches improve segmentation accuracy by leveraging multi-modal cues, they often lack fine-grained alignment between 2D and 3D features, leading to suboptimal fusion effectiveness.

Recent studies explore cross-domain feature alignment and joint representation learning to better bridge the gap between modalities. For example, fusion strategies based on shared latent spaces, feature correlation learning, or attention-guided integration aim to dynamically weigh contributions from each modality. Despite these advances, achieving accurate point-to-pixel correspondence and mitigating the structural inconsistencies between unstructured point clouds and regular image grids remains an open problem, especially in large-scale and noisy urban scenes.

### 2.3 Cross-Modal attention and feature enhancement

Attention mechanisms have gained prominence for their ability to model long-range dependencies and capture complex relationships in both intra-modal and cross-modal learning. Self-attention, as employed in transformer-based architectures, enables point cloud networks to consider global context by computing pairwise interactions among all points [24]. This is particularly valuable in urban scenes, where object instances may be large, and spatial dependencies extend across wide areas.

Cross-modal attention further extends this idea by enabling interactive learning between different modalities. In multi-modal segmentation, cross-attention modules allow point cloud features to attend to corresponding image features and vice versa, dynamically fusing texture and geometric cues. Methods like FusionTransformer [25] have successfully applied such mechanisms in indoor environments, demonstrating that hybrid attention can significantly enhance segmentation accuracy.

However, most existing cross-modal attention strategies have been developed for relatively small-scale datasets with well-aligned modalities, such as RGB-D indoor scans. When applied to urban-level outdoor scenes, these approaches face challenges from scale variation, partial data overlap, and noise in both modalities. Furthermore, projection from 3D to 2D may cause irreversible information loss, limiting the effectiveness of downstream attention mechanisms.

### 2.4 Motivation for RPINet

Considering the limitations in current approaches, RPINet is proposed to tackle the challenges of urban-scale multi-modal semantic segmentation. First, we adopt Gaussian splatting during the 3D-to-2D projection to preserve local geometric continuity and reduce information loss. Second, we design a powerful point cloud feature extraction pipeline that combines DBSCAN-based clustering for region organization, PointNet++ for local geometric encoding, transformers for global contextual modeling, and graph convolutional networks (GCN) for relational refinement. While more recent architectures such as Point Transformer [24] offer strong standalone performance, their global self-attention mechanisms overlap significantly with the dedicated Transformer module already present in our pipeline. Consequently, PointNet++ is deliberately retained as a lightweight and well-suited local encoder, avoiding architectural redundancy and preserving inference efficiency. This allows RPINet to capture both fine details and large-scale structure across the urban scene.

To fully exploit the complementary nature of 2D and 3D data, RPINet integrates a hybrid cross-modal attention mechanism, enabling interactive learning between 2D remote sensing features and 3D point cloud representations. Finally, a unified loss function is introduced, balancing information preservation, segmentation accuracy, and spatial smoothness.

By holistically addressing the challenges of large-scale data handling, multi-modal feature fusion, and context-aware segmentation, RPINet sets a new direction for robust, accurate, and efficient urban-scale point cloud semantic segmentation.

## 3 Methodology

### 3.1 Overall framework

**Fig 2:**
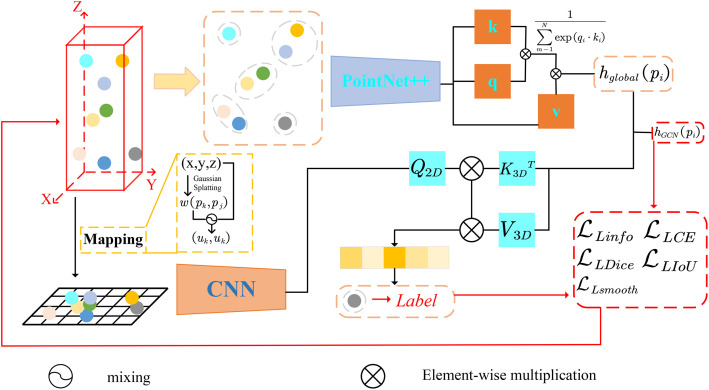
Method. Our method employs a dual-branch modeling approach using point cloud data. It fully utilizes the 2D features of 3D point clouds in remote sensing data and integrates the inherent information of the 3D point clouds for feature fusion. Multiple loss functions are used to ensure information alignment, ultimately achieving accurate target classification. Any illustrative point-cloud or label snippets shown in this schematic are adapted from the SensatUrban dataset by Hu et al. [1] and used under the MIT License from the official SensatUrban repository.

RPINet employs a dual-branch architecture to integrate 3D point cloud data with 2D remote sensing imagery for semantic segmentation. Let $P=\left(p_{k},c_{k}\right),{k=1}^{N}$ be the input point cloud with *N* points, where each point $p_{k}=(x_{k},y_{k},z_{k})\in {\mathbb{R}}^{3}$ is a 3D coordinate and $c_{k}=(r_{k},g_{k},b_{k})$ is the corresponding color (RGB) information. The goal is to predict a semantic label $\hat{\ell }k$ for each point *p*_*k*_. We formulate the segmentation task as learning a function D(P∣θ), parametrized by θ, that outputs a probability distribution over *C* semantic classes for each point. The predicted label for point *p*_*k*_ is then ${\hat{\ell }}_{k}=\arg\max_{c}D_{k,c}(P|\theta )$, where $D_{k,c}$ is the predicted probability of class *c* for point *k* (Fig 2).

To achieve this, RPINet processes the data through two coordinated branches: a Remote-Image branch that focuses on texture and appearance features in 2D, and a Point-Cloud branch that emphasizes 3D geometric features. The Remote-Image (RI) branch projects the point cloud onto a 2D image plane (using known sensor calibration or a top-down projection) and applies image processing techniques to enhance and extract multi-scale features. The Point-Cloud (PC) branch operates directly on the 3D points, performing clustering, local-to-global feature extraction (PointNet++ style encoding, transformer-based attention, and graph convolution), as well as adaptive sampling and multi-scale processing to capture geometric details at various scales. The high-level features from both branches are then fused via an attention-guided module that learns to highlight relevant intra-modal and cross-modal information. Finally, a segmentation head produces the per-point label probabilities. We also design a multi-term loss function to jointly optimize semantic accuracy and structural consistency. The following subsections describe each component in detail, with rigorous mathematical formulation and justification.

### 3.2 Remote-image branch: Gaussian splatting and 2D feature extraction

#### 3.2.1 3D-to-2D projection with Gaussian splatting‌‌.

To leverage 2D convolutional networks on irregular point clouds, we first project the 3D points onto a 2D grid, creating a pseudo-image. A naive projection can cause aliasing and information loss, especially in sparse regions. We address this by applying Gaussian splatting as a pre-processing step, which smooths the point cloud and distributes each point’s influence to its neighbors before projection. For any point $p_{k}=(x_{k},y_{k},z_{k})\in P$ and a neighboring point *p*_*j*_ = (*x*_*j*_, *y*_*j*_, *z*_*j*_), we define the Gaussian weight (influence) of *p*_*k*_ on *p*_*j*_ as:


$$\begin{array}{c} w(p_{k},p_{j})=\exp(-\frac{(x_{k}-x_{j}{)}^{2}+(y_{k}-y_{j}{)}^{2}+(z_{k}-z_{j}{)}^{2}}{2\sigma^{2}}), \end{array}$$ (1)

where σ is the standard deviation of the Gaussian kernel controlling the spatial extent of the influence. This weight decays exponentially with Euclidean distance, so closer points have a larger mutual influence. Intuitively, Gaussian splatting performs a locally weighted smoothing of the point cloud: each point “spreads” a fraction of its value (position or features) to nearby points, akin to distributing mass in a continuous density map. This process reduces noise and fills small gaps by ensuring that isolated or sparse points contribute to the representation of their vicinity, thereby enhancing local continuity.

Using the weights from (1), we adjust each point’s position toward its neighbors. For a given point *p*_*k*_ and one of its neighbors *p*_*j*_, the neighbor can be shifted slightly toward *p*_*k*_ by:


$$\begin{array}{c} p_{j}^{\prime }=p_{j}+w(p_{k},p_{j})(p_{k}-p_{j}), \end{array}$$ (2)

where $p_{j}^{\prime }$ is the updated neighbor position. In practice, we iteratively or simultaneously apply such shifts for all points in a local neighborhood, effectively smoothing the point cloud geometry. The influence of *p*_*k*_ on *p*_*j*_ is strongest when *p*_*j*_ lies very close to *p*_*k*_, and diminishes for distant points. As a result, Gaussian splatting preserves fine local structures while mitigating outliers: points within a tight cluster move closer together (denoising small isolated noise), and small gaps are bridged by intermediate influence, but large-scale geometry remains unchanged since distant points have negligible effect on each other. After Gaussian splatting, the point distribution is more uniform and continuous, which is beneficial for the subsequent projection.

#### 3.2.2 Quantized projection to image plane.

Once the point cloud has been smoothed, we project the 3D points onto a 2D plane to generate a rasterized representation. Given an appropriate viewpoint (e.g., an overhead orthographic view for aerial data or a perspective view with known camera intrinsics/extrinsics), we define a projection function. For simplicity, assuming an orthonormal projection, we map coordinates by quantizing the *x* and *y* values of each point to integer pixel coordinates:


$$\begin{array}{cc} \left(u_{k},v_{k}\right) & =\pi (p_{k})\\ & =(\lfloor x_{k}/s\rfloor ,\lfloor y_{k}/s\rfloor ), \end{array}$$ (3)

where (*u*_*k*_, *v*_*k*_) are the 2D pixel coordinates of point *p*_*k*_ on the image plane, and *s* is a quantization factor (grid resolution) that determines the size of each pixel. This operation effectively bins points into pixels: all points falling into the same (⌊x/s⌋,⌊y/s⌋) bin are mapped to the same image pixel. In a more general camera model, π(·) would incorporate perspective projection equations using calibration parameters, but the end result is the same—a set of image coordinates corresponding to the 3D points. We retain the association between each point *p*_*k*_ and its pixel (*u*_*k*_, *v*_*k*_) for later fusion. The projection compresses the 3D structure onto a 2D grid while, thanks to Gaussian splatting, preserving neighborhood relationships. Specifically, the Gaussian smoothing ensures that if a 3D structure is somewhat sparse, its projection will still carry a continuous intensity or feature distribution rather than isolated pixels, thereby reducing information loss during quantization.

The projection yields an initial 2D image *I* (with channels for accumulated color or density) of size *H* × *W* pixels covering the area of interest. Each pixel in *I* encodes the presence (and possibly aggregated color/features) of points in the corresponding 3D column of space. This forms the input to the image processing stage of the RI branch.

#### 3.2.3 Multi-scale CNN feature extraction.

With the 2D projection *I* in hand, we employ a 2D convolutional neural network to extract high-level features that capture textures, edges, and contextual cues from the remote sensing image. We denote the image feature extraction as:


$$\begin{array}{c} F^{\left(I\right)}=\Phi_{\text{CNN}}(I), \end{array}$$ (4)

where $F^{\left(I\right)}\in {\mathbb{R}}^{H\times W\times C_{1}}$ is the resulting feature map (with *C*_1_ channels) produced by the CNN $\Phi_{\text{CNN}}$. This CNN can be a standard encoder (e.g., ResNet or U-Net backbone) that progressively extracts features from low-level (edges, textures) to high-level (object-level context). We adopt a multi-scale feature extraction strategy to ensure both local details and global contexts are captured. In practice, this can be implemented by a feature pyramid network or by using convolution layers with different receptive fields. We obtain feature maps at multiple scales (levels of resolution) and combine them to form a comprehensive representation:


$$\begin{array}{c} F_{\text{fusion}}^{\left(I\right)}=\sum_{\ell =1}^{L}w_{\ell },F_{\ell }^{\left(I\right)}, \end{array}$$ (5)

where $F_{\ell }^{\left(I\right)}$ is the feature map extracted at scale (or level) ℓ, and $w_{\ell }$ is a learned weight reflecting the importance of that scale. The summation in (5) indicates a weighted fusion (or it could be a concatenation followed by a 1x1 convolution) of multi-scale feature maps, yielding a final 2D feature representation $F_{\text{fusion}}^{\left(I\right)}$ that contains information from coarse (contextual) to fine (detailed) levels. By doing so, the RI branch ensures that features like building edges or tree textures (fine details) as well as broader layout cues (e.g., a cluster of pixels forming a large roof or road, giving context) are both preserved. The output of this branch is a dense feature map covering the entire projected scene. This Remote-Image branch output will later be aligned and fused with the point cloud branch output. Crucially, the RI branch provides rich color and texture cues that complement the geometric features from the point cloud branch, helping to distinguish objects that might be geometrically similar but visually different (for instance, a tree vs. a lamp post might have similar shapes but very different image textures).

### 3.3 Point-cloud branch: Multi-scale geometric feature learning

In parallel to the RI branch, the Point-Cloud branch processes the 3D data directly to extract robust geometric features. This branch is composed of several modules that together learn point representations encoding local geometry, global structure, and inter-point relationships. We denote the final output of this branch as $F^{\left(P\right)}={h_{i}^{\left(3D\right)}}_{i=1}^{N}$, where $h_{i}^{\left(3D\right)}\in {\mathbb{R}}^{C_{2}}$ is the feature vector learned for point *p*_*i*_. Below we detail each module in this branch and how it contributes to semantic segmentation.

#### 3.3.1 Geometric clustering for regional features.

Instead of treating every point in isolation, we first organize the point cloud into meaningful clusters that reflect local surfaces or objects. Clustering reduces data complexity and provides a structural context for each point. We employ a density-based clustering (DBSCAN) to group points that are spatially close and densely distributed. Formally, for DBSCAN we define a neighborhood radius ε and minimum points *m*. A point *p*_*i*_ is considered a core point if it has at least *m* neighbors within distance ε. DBSCAN then defines an equivalence relation on points: *p*_*i*_ ∼ *p*_*j*_ if there exists a chain of points $p_{k_{0}}=p_{i},p_{k_{1}},\ldots ,p_{k_{L}}=p_{j}$ such that each consecutive pair lies within ε of each other. All points related by ∼ form a cluster *C*_*k*_. This identifies contiguous high-density regions (e.g., points on the same facade or ground patch). To capture larger-scale groupings, we also construct a graph *G*=(*V*,*E*) over clusters or points, where each node represents a point (or an initial cluster) and edges e=(i,j)∈E connect points that are in proximity or have similar attributes (color, normal, etc.) even if they belong to different DBSCAN clusters. For example, we add an edge between *p*_*i*_ and *p*_*j*_ if $||p_{i}-p_{j}||<\tau_{d}$ (distance threshold) or if their feature similarity (e.g., normal vector difference or color difference) is above a threshold $\tau_{f}$. We can then apply graph-based clustering (such as spectral clustering or community detection on *G*) to merge the DBSCAN clusters into larger super-clusters or supervoxels $C^{\prime }u$. The result of this two-stage clustering is a set $𝒞=C_{1},C_{2},\ldots ,C_{K}$ of clusters (ranging from small, dense regions to broader structures). We assign each point *p*_*i*_ a cluster label c(i)∈1,…,K indicating which cluster it belongs to. We also compute a cluster-level feature descriptor for each cluster *C*_*k*_, for instance by averaging or pooling the point features in that cluster:


$$\begin{array}{c} h_{C_{k}}=\frac{1}{\left|C_{k}\right|}\sum_{p_{i}\in C_{k}}f(p_{i}), \end{array}$$ (6)

where *f*(*p*_*i*_) could be the initial feature of point *p*_*i*_ (such as a positional encoding or color) or a learned intermediate feature. These cluster descriptors $h_{C_{k}}$ provide a compact representation of region-wise properties. The clustering step benefits segmentation by capturing regional context: points within the same cluster likely belong to the same object or surface (hence share a label), so the model can use the cluster identity as a strong cue for segmentation consistency. Moreover, clustering helps focus the later stages of the network on meaningful groups rather than isolated points, improving efficiency and robustness to noise (outliers may fall into small clusters or remain unclustered and can be handled separately).

#### 3.3.2 Local feature encoding (PointNet++).

It is important to note that PointNet++ is adopted here in a specific and limited role: it serves solely as a local neighborhood feature encoder, not as the primary backbone of the network. Global contextual modeling is explicitly delegated to the dedicated self-attention Transformer in Module 3, while relational refinement is handled by the GCN in Module 4. Replacing PointNet++ with a globally-aware architecture such as Point Transformer [24] or PointNeXt would introduce functional redundancy with the downstream Transformer module, increasing model complexity without commensurate benefit. PointNet++ is therefore deliberately chosen as a lightweight and effective component for this local encoding stage, consistent with our design goal of maintaining inference efficiency. Next, we extract point-wise features that capture fine-grained local geometry. Drawing inspiration from PointNet++’s set abstraction, we consider each point *p*_*i*_ and its neighborhood *N*(*i*) (for example, all points within radius *r* of *p*_*i*_, or the *k* nearest neighbors). We then apply a small point-based neural network (MLP) on each neighbor’s relative position and initial feature (e.g., relative coordinates and color) and aggregate these to form a local descriptor for *p*_*i*_. Formally, let g(·) be an MLP that maps a concatenation of a neighbor’s relative coordinates and features to a latent representation. We compute:


$$\begin{array}{c} h_{i}^{\text{local}}=\max_{p_{j}\in N(i)}g([p_{j}-p_{i},f(p_{j})]), \end{array}$$ (7)

where [·,·] denotes concatenation of the geometric offset *p*_*j*_ − *p*_*i*_ and the neighbor’s feature *f*(*p*_*j*_) (which initially could be just color or height, and later can be replaced by intermediate learned features), and the max operator is applied element-wise to the set of neighbor feature outputs to yield an invariant summary (one could also use average pooling). The result $h_{i}^{\text{local}}$ is a fixed-length feature vector encoding the shape of the local neighborhood around *p*_*i*_ (e.g., flat surface vs. edge vs. corner) and the distribution of any attributes. This is analogous to the local feature extraction in PointNet++  which captures fine geometry like curvature or roughness around each point. By computing $h_{i}^{\text{local}}$, the network gains an understanding of small-scale structures, which is crucial for distinguishing adjacent classes (for instance, ground vs. curb vs. wall might differ in the curvature or normal variation in their local neighborhoods).

#### 3.3.3 Global context encoding (self-attention transformer).

While local features are important, points in large-scale scenes can be far apart yet related (e.g., two walls on opposite sides of a street are both walls and part of the same street scene). To capture long-range dependencies and global context, we feed the point features into a self-attention module inspired by transformer architectures. We treat the set of point features $h_{i}^{\text{local}},{i=1}^{N}$ as a sequence of “tokens.” For each point *i*, we compute query, key, and value vectors via learnable linear projections of the local feature:


$$\begin{array}{c} q_{i}=W_{Q},h_{i}^{\text{local}},\\ k_{i}=W_{K},h_{i}^{\text{local}},\\ v_{i}=W_{V},h_{i}^{\text{local}}, \end{array}$$ (8)

where $W_{Q},W_{K},W_{V}\in {\mathbb{R}}^{d\times d\text{in}}$ are projection matrices (with *d*in the dimension of *h*^local^*i* and *d* the chosen dimensionality of the queries/keys). The self-attention mechanism allows each point *i* to attend to all other points *j* through the compatibility of their query and key. We compute attention weights and updated features as:


$$\begin{array}{cc} \alpha_{ij} & =\frac{\exp((q_{i}\cdot k_{j})/\sqrt{d})}{\sum_{m=1}^{N}\exp((q_{i}\cdot k_{m})/\sqrt{d})},\\ \;h_{i}^{\text{global}} & =\sum_{j=1}^{N}\alpha_{ij},v_{j}. \end{array}$$ (9)

Here $\alpha_{ij}$ represents the attention weight that point *i* assigns to point *j*; it is essentially a learned affinity between points *i* and *j* normalized across all *j*. The updated feature $h_{i}^{\text{global}}$ is a weighted sum of value vectors from all points, so it integrates information from the entire point cloud, with more focus on points that are deemed relevant to *i*. In practice, we use multi-head self-attention: the projection in (8) and computation in (9) are done *H* times (with independent $W_{Q}^{h},W_{K}^{h},W_{V}^{h}$ for head h=1…H and smaller *d* per head), and the results are concatenated to form a rich global feature for each point. The transformer’s self-attention endows each point’s representation $h_{i}^{\text{global}}$ with awareness of the overall scene layout and long-range interactions. For example, a point on a car will receive contextual information from other points on the same car (to understand the extent of that object) and even from points on the road underneath or buildings nearby, as the model learns to attend to relevant contexts for segmentation. This global feature helps disambiguate cases where local geometry alone is insufficient (many objects might have planar surfaces locally, but their global arrangement or co-occurrence helps identify what they are).

#### 3.3.4 Relational refinement with graph convolution.

After obtaining global context features, we further refine the point representations by incorporating local relational structure explicitly, using Graph Convolutional Networks (GCNs). While the self-attention treated all point pairs with equal potential, a GCN can enforce that only points with actual connections (e.g., spatial neighbors or within the same cluster) exchange information, thus reinforcing locality and smoothing within object regions. We construct an adjacency relation *A*_*ij*_ for points, for example *A*_*ij*_ = 1 if point j∈N(i) (a neighbor in the point cloud within radius or KNN) or if *c*(*j*)=*c*(*i*) (points belong to the same cluster from step 1), and *A*_*ij*_ = 0 otherwise. A simple first-order graph convolution update for each point *i* is then:


$$\begin{array}{c} h_{i}^{\text{GCN}}=\sigma (W_{0},h_{i}^{\text{global}}+W_{1}\sum_{j\in N(i)}\frac{1}{\left|N(i)\right|}h_{j}^{\text{global}}), \end{array}$$ (10)

where *W*_0_, *W*_1_ are learnable weight matrices and σ(·) is a nonlinear activation (e.g., ReLU). In this formulation, each point *i* updates its feature by combining its current feature $h_{i}^{\text{global}}$ with an average of the features of its neighbors j∈N(i). This is essentially a graph smoothing or averaging that promotes similarity among connected points. More advanced GCN variants could weight each neighbor in the sum by a function of $h_{j}^{\text{global}}$ and $h_{i}^{\text{global}}$ or by edge attributes, but the essence remains: points that are spatially or semantically connected (like belonging to the same cluster or object) will align their features. This graph convolutional refinement encourages consistency: if a point is on a planar roof and its neighbors are also on that roof, their features after GCN will become more similar, making it easier for the classifier to assign them the same label. Conversely, at object boundaries, neighbors might belong to different clusters or be excluded from the adjacency, allowing discontinuities to remain where appropriate. After the GCN step, we obtain the refined feature $h_{i}^{\text{GCN}}$ for each point *p*_*i*_. At this stage, each $h_{i}^{\text{GCN}}$ encodes multi-scale information: (i) the local neighborhood geometry (from PointNet++), (ii) the global context (from transformer attention), and (iii) the relational context enforcing that points on the same structure have similar representations (from GCN). We denote this entire point feature extraction process as a function $h_{i}^{GCN}=F_{3D}(p_{i},\{p_{j}\})$,parameters), summarizing that for each point the branch produces a *C*_2_-dimensional feature vector.

#### 3.3.5 Adaptive point sampling (efficiency and focus).

Processing every point in a large-scale cloud (with millions or more points) is computationally expensive and sometimes redundant. We incorporate an adaptive sampling strategy to select a subset of points that are most informative for segmentation, reducing the computational burden while maintaining performance. The idea is to reduce the number of points from *N* to *M* (*M* < *N*) for certain expensive operations, focusing on critical areas (e.g., boundaries, distinct structures) and down-sampling repetitive or homogeneous regions.

One approach is importance-based sampling. We assign each point *p*_*i*_ an importance score *I*(*p*_*i*_) that reflects how much that point contributes to segmentation accuracy. The score can be hand-crafted (e.g., higher for points with large curvature, significant height difference from neighbors, or near color edges in the image) or learned (e.g., using a small network that predicts uncertainty or feature magnitude). We then choose the top *M* points with the highest scores. Mathematically, if S⊂1,…,N is the index set of selected points (with |*S*| = *M*), we want


$$\begin{array}{c} S=\arg\max_{|S|=M}\sum_{i\in S}I(p_{i}), \end{array}$$ (11)

meaning we aim to maximize the total importance of retained points. In practice, solving (11) exactly is combinatorial, so we use greedy heuristics or thresholding on *I*(*p*_*i*_) (e.g., pick all points above a certain importance value). This tends to keep points on edges or uncommon classes (high *I*) while dropping points in flat, uniformly labeled areas (low *I*), thus preserving segmentation-critical structures.

Another approach is reinforcement learning-based sampling. We can frame point selection as a sequential decision process where a policy π chooses points to include such that a reward (e.g., segmentation accuracy or IoU on a validation set) is maximized. A neural network can be trained to output a probability of selecting each point, and a reinforcement learning algorithm (like policy gradients) updates π based on the reward. For example, an agent could learn to mimic farthest point sampling (to ensure coverage) but also to focus on high-curvature areas (to ensure detail). A differentiable approximation of sampling (such as the SampleNet approach) creates a soft selection by generating new points $p_{j}^{\sim }$ as weighted combinations of original points:


$$\begin{array}{c} p_{j}^{\sim }=\sum_{i=1}^{N}w_{ij},p_{i},\;j=1,\ldots ,M, \end{array}$$ (12)

with weights *w*_*ij*_ that form a sparse selection matrix (each $p_{j}^{\sim }$ ideally equals one of the input points if one weight is 1 and others 0). This can be trained end-to-end by backpropagating a surrogate loss that encourages picking points that lead to good segmentation. Regardless of the mechanism, the output of adaptive sampling is a reduced set $P_{S}=p_{i}:i\in S$ that is passed through the feature extraction pipeline (or one could apply sampling at multiple stages, such as at each layer of a hierarchical set abstraction, described next). By focusing computation on the most informative points, the network can allocate more capacity to complex regions. Moreover, sampling can act as a form of data augmentation and regularization: if at each epoch a slightly different subset of points is chosen, the model is exposed to varied inputs and must rely on robust features rather than overfit to every single point. Our adaptive sampling thus strikes a balance between efficiency and accuracy, ensuring that minimal critical information is lost when reducing point density.

#### 3.3.6 Multi-scale feature pyramid.

Urban scenes contain structures of vastly different scales, from small objects like street signs to large expanses like roads and buildings. To achieve scale-invariant segmentation, we adopt a multi-scale processing strategy for the point cloud akin to an encoder-decoder pyramid. We generate a hierarchy of point sets with decreasing resolution: *P*^(0)^ = *P* is the original set of *N* points, and for each level ℓ=0,1,…,L−1 we sample a subset $P^{\left(\ell +1\right)}\subset P^{\left(\ell \right)}$ such that $\left|P^{\left(\ell +1\right)}|<|P^{\left(\ell \right)}\right|$. The sampling can be random, via farthest point sampling (FPS), or using the aforementioned adaptive sampling. Points in $P^{\left(\ell +1\right)}$ can be thought of as representative points (or centroids) for neighborhoods in $P^{\left(\ell \right)}$. For each representative point $q\in P^{\left(\ell +1\right)}$, we define its receptive field in the lower level as the set of points within a radius $r_{\ell }$: $p\in P^{\left(\ell \right)}:|p-q|<r_{\ell }$. We then aggregate features from level ℓ to compute the feature for *q* at level ℓ+1. For example, using a PointNet-based aggregator:


$$\begin{array}{c} h_{q}^{\left(\ell +1\right)}=\max_{p\in P^{\left(\ell \right)},|p-q|<r_{\ell }}\Psi^{\left(\ell \right)}(h_{p}^{\left(\ell \right)},p-q), \end{array}$$ (13)

where $h_{p}^{\left(\ell \right)}$ is the feature of point *p* at level ℓ (with *h*^(0)^*p* initialized as some function of raw coordinates and color, or after initial local feature encoding), and $\Psi^{\left(\ell \right)}$ is an MLP that combines a point’s current feature with its relative position to the representative *q*. The max (or average) pooling yields the feature $h_{q}^{\left(\ell +1\right)}$ that represents all points in *q*’s neighborhood. Equation (13) is applied iteratively from ℓ=0 up to *L*, yielding progressively coarser but more context-encompassing features. By level *L*, we might have only a handful of points (superpoints) summarizing the entire scene with very large receptive fields.

After obtaining multi-scale features, we can fuse features across scales to enrich the representation of each original point with both fine and coarse information. One approach is to propagate the coarse features back to each original point (a decoder or upsampling step). For each point $p_{i}=p_{i}^{\left(0\right)}$ in the original cloud, we find its corresponding ancestor $p_{i}^{\left(1\right)}$ in *P*^(1)^ (the representative that covered *p*_*i*_’s neighborhood), then that point’s ancestor $p_{i}^{\left(2\right)}$ in *P*^(2)^, and so on up to $p_{i}^{\left(L\right)}$. We then concatenate all these features:


$$\begin{array}{c} h_{i}^{\text{multi}}=[h_{p_{i}}^{\left(0\right)},h_{p_{i}}^{\left(1\right)},\ldots ,h_{p_{i}}^{\left(L\right)}], \end{array}$$ (14)

where $h_{p_{i}}^{\left(\ell \right)}$ denotes the feature of the ancestor of point *p*_*i*_ at level ℓ. In practice, a learned fusion (such as summing after projecting to a common dimension, or using attention across scales) can be applied instead of simple concatenation. The result $h_{i}^{\text{multi}}$ is a multi-scale feature descriptor for point *p*_*i*_ that encodes information from a very local scale (level 0, possibly after the GCN refinement) up to a global scale (level *L*). This is highly beneficial for segmentation: small objects or details (e.g., a traffic sign) will primarily be recognized from the fine-scale component of $h_{i}^{\text{multi}}$, whereas large structures (like terrain vs. building) will be distinguished by coarse-scale components. The combination allows the network to handle scale variability: for example, what constitutes a “wall” can be a few meters or tens of meters long, but the multi-scale feature can represent both a segment of wall and the fact that it is part of a larger building. Empirically, multi-scale fusion improves the model’s ability to accurately segment both small and large objects, leading to more coherent and complete scene interpretations.

#### 3.3.7 Point-image feature alignment for fusion.

The culmination of the point-cloud branch is a rich set of features $h_{i}^{\left(3D\right)}$ for each point *p*_*i*_. Before fusing with image features, we ensure that these 3D features are properly aligned with their corresponding image locations. Since we projected each point onto the image plane earlier (Equation (3)), we know the pixel coordinate (*u*_*i*_, *v*_*i*_) associated with point *p*_*i*_. We leverage this correspondence to pair each point’s 3D feature with the 2D feature at the same location. Let $F_{\text{fusion}}^{\left(I\right)}(u,v)$ be the 2D fused feature map from the RI branch (Eq. (5)). For each point *p*_*i*_, we retrieve the image feature vector at its pixel: $g_{i}=F_{\text{fusion}}^{\left(I\right)}(u_{i},v_{i})$. Now we form an initial fused representation for point *i* by concatenation:


$$\begin{array}{c} z_{i}^{\text{init}}=[h_{i}^{\left(3D\right)},g_{i}]\in {\mathbb{R}}^{C_{2}+C_{1}}, \end{array}$$ (15)

which simply attaches the 2D modality features to the 3D modality features for the same physical point. In our implementation, we typically project both $h_{i}^{\left(3D\right)}$ and *g*_*i*_ into a common size and sum or concatenate them as appropriate. The key point is that by using the known projection mapping π, we achieve a one-to-one point-to-pixel correspondence—each point feature knows exactly which image feature comes from the same location in the world. This eliminates any need for ambiguous nearest-neighbor matching between modalities and ensures that fusion is geometrically meaningful. For instance, a point on a tree will be paired with the exact image pixel of that tree’s canopy or trunk, so the green texture from the image is directly fused with the 3D shape from the point. This alignment sets the stage for the subsequent cross-modal fusion module, which will more deeply integrate these features. The initial concatenation $z_{i}^{\text{init}}$ could also be processed through a small network Ψ(·) to begin mixing the modalities (e.g., a linear layer or two to produce a fused vector $h_{i}^{\text{fusion}}$), or structured in a grid (for example, forming a 2D grid of fused features if we rasterize the point cloud) to apply further convolution. In our design, we proceed with an attention-based fusion described next.

### 3.4 Cross-modal attention fusion and segmentation

After both branches have produced their feature representations, we employ an attention-guided fusion module to effectively merge the 2D and 3D information. The fused features are then passed to the segmentation head to predict semantic labels. A core challenge in fusion is that the image branch yields a structured feature map $F_{\text{fusion}}^{\left(I\right)}(u,v)$ over pixels, whereas the point cloud branch yields features $h_{i}^{\left(3D\right)}$ associated with irregular point locations. We address this by the alignment step above, effectively converting the fused point features $z_{i}^{\text{init}}$ into a unified set of features where each element corresponds to a point (and its pixel). We can denote the set of fused point-wise features as $𝒵=z_{i},{i=1}^{N}$, where $z_{i}\in {\mathbb{R}}^{D}$ (with $D=C_{1}+C_{2}$ after concatenation or a reduced dimension if we project them to a common size).

However, not every point in the original cloud will have a valid corresponding pixel (some might project to the same pixel or outside the image bounds if using perspective). For simplicity, assume *M* distinct fused features remain after handling any collisions (one could combine points that fall in the same pixel by averaging their features, or just treat one and ignore others if very dense). We reshape or index these fused features as matrices suitable for attention. Let $F_{2D}^{\prime }\in {\mathbb{R}}^{M\times d_{f}}$ and $F_{3D}^{\prime }\in {\mathbb{R}}^{M\times d_{f}}$ be two feature sets of dimension *d*_*f*_ for each of the *M* correspondences, derived from the 2D and 3D branches respectively. In the simplest case, $F_{2D}^{\prime }$ could be *g*_*i*_ and $F_{3D}^{\prime }$ could be $h_{i}^{\left(3D\right)}$, possibly after up-projecting to length *d*_*f*_. In practice, we use learnable embedding functions $\phi_{2D}$ and $\phi_{3D}$ to ensure both modalities’ features live in the same feature space:


$$\begin{array}{cc} F_{2D}^{\prime } & =\phi_{2D}(F_{\text{fusion}}^{\left(I\right)})\in {\mathbb{R}}^{M\times d_{f}},\\ F_{3D}^{\prime } & =\phi_{3D}(h_{i}^{\left(3D\right)})\in {\mathbb{R}}^{M\times d_{f}}. \end{array}$$ (16)

Here $\phi_{2D}$ can be implemented as a 1 × 1 convolution or a small CNN that takes the 2D feature map and produces a *d*_*f*_-dimensional feature for each pixel (which we then sample at the *M* point-pixel locations), and $\phi_{3D}$ can be a fully-connected layer or MLP applied to each 3D point feature. Now $F_{2D}^{\prime }=[f_{1}^{\prime },f_{2}^{\prime },\ldots ,f_{M}^{\prime }{]}_{2D}$ and $F_{3D}^{\prime }=[f_{1}^{\prime },f_{2}^{\prime },\ldots ,f_{M}^{\prime }{]}_{3D}$ correspond in indices (i.e., $f_{i,2D}^{\prime }$ and $f_{i,3D}^{\prime }$ are the features for the same point-pixel *i*).

We propose a hybrid attention mechanism that includes both self-attention within each modality and cross-attention between modalities. First, to refine each modality’s features individually (intra-modal interaction), we apply multi-head self-attention on $F_{2D}^{\prime }$ and on $F_{3D}^{\prime }$ separately. Using the standard notation from transformers, for a feature matrix $F\in {\mathbb{R}}^{M\times d_{f}}$, we form queries $Q=FW_{Q}$, keys $K=FW_{K}$, and values $V=FW_{V}$ with learned projection matrices $W_{Q},W_{K},W_{V}\in {\mathbb{R}}^{d_{f}\times d_{f}}$. Then the self-attention operator is:


$$\begin{array}{c} \text{SelfAttn}(F)=softmax(\frac{QK^{T}}{\sqrt{d_{f}}})V, \end{array}$$ (17)

which yields an output of the same shape *M* × *d*_*f*_. We apply this to each modality:


$$\begin{array}{c} \tilde{F}2D=\text{SelfAttn}(F{2D}^{\prime }),\\ \tilde{F}3D=\text{SelfAttn}(F{3D}^{\prime }). \end{array}$$ (18)

This operation lets features within the 2D map inform each other (capturing 2D context among pixels corresponding to points) and similarly lets 3D point features refine each other by attention (capturing 3D context, similar to what was done in the point branch but now in the fused feature space).

Next, for cross-modal attention, we allow features from one modality to attend to the other. Specifically, we compute an attention from 2D to 3D and another from 3D to 2D. For the 2D-to-3D attention, we treat $F_{2D}^{\prime }$ as the query source and $F_{3D}^{\prime }$ as the key-value source:


$$\begin{array}{c} \text{CrossAttn}2D\leftarrow 3D=softmax(\frac{Q_{2D}K_{3D}^{T}}{\sqrt{d_{f}}})V_{3D}, \end{array}$$ (19)

where $Q_{2D}=F_{2D}^{\prime }W_{Q}^{\left(c\right)}$ and $K_{3D}=F_{3D}^{\prime }W_{K}^{\left(c\right)}$, $V_{3D}=F_{3D}^{\prime }W_{V}^{\left(c\right)}$ are projections for cross-attention (with their own learned parameters *W*^(*c*)^). This yields a feature set denoted $CA_{2D\leftarrow 3D}$, meaning “the information in 3D features as viewed by (attended by) the 2D queries.” Intuitively, each pixel-based feature queries the point cloud features to find relevant complementary information. Similarly, for 3D-to-2D:


$$\begin{array}{c} \text{CrossAttn}3D\leftarrow 2D=softmax(\frac{Q_{3D}K_{2D}^{T}}{\sqrt{d_{f}}})V_{2D}, \end{array}$$ (20)

with $Q_{3D}=F_{3D}^{\prime }W_{Q}^{\left(c^{\prime }\right)}$ and $K_{2D}=F_{2D}^{\prime }W_{K}^{\left(c^{\prime }\right)}$, $V_{2D}=F_{2D}^{\prime }W_{V}^{\left(c^{\prime }\right)}$, producing features $CA_{3D\leftarrow 2D}$. These cross-attention steps enable interactive learning: e.g., if a certain 3D point feature is ambiguous, attending to the 2D features might clarify its identity (the 3D feature for a point on a tree can attend to the green color in the 2D feature at that location, strengthening the indication that it’s vegetation). Conversely, a 2D pixel feature that might be unclear due to shadow or image blur can attend to the 3D geometry to understand that it’s part of a building or ground.

We then aggregate the results of intra- and inter-modal attention. Let ${\tilde{F}}_{2D},{\tilde{F}}_{3D}$ be the self-attended features from (18), and $CA_{2D\leftarrow 3D},CA_{3D\leftarrow 2D}$ be the cross-attended features from (19) and (20). We combine them as:


$$\begin{array}{cc} F_{\text{fusion}}(i)= & \gamma_{1},\tilde{F}2D(i)+\gamma_{2},\tilde{F}3D(i)+\gamma_{3},\\ & CA_{2D\leftarrow 3D}+\gamma_{4},CA_{3D\leftarrow 2D}, \end{array}$$ (21)

for each correspondence index i=1,…,M. Here $\gamma_{1},\gamma_{2},\gamma_{3},\gamma_{4}$ are learnable scalar weights that balance the contribution of each component. The final fused feature *F*_fusion_(*i*) thus contains 2D information (refined within 2D), 3D information (refined within 3D), and the exchanged information between 2D and 3D. In practice, this fused feature set $F_{\text{fusion}}=F_{\text{fusion}}(i),{i=1}^{M}$ can be viewed as a set of *M* fused point/pixel features each of dimension *d*_*f*_. We then feed *F*_fusion_ into the segmentation head.

#### 3.4.1 Segmentation head and output.

The segmentation head is a classifier that maps each fused feature *F*_fusion_(*i*) to a distribution over the *C* semantic classes. This can be implemented by a small fully-connected network (two or three layers) or even a single linear layer followed by softmax. Formally, let $S:{\mathbb{R}}^{d_{f}}\to {\mathbb{R}}^{C}$ denote the segmentation head mapping a fused feature to class logits. We apply it to each fused feature vector:


$$\begin{array}{c} {\hat{y}}_{i}=S(F_{\text{fusion}}(i)), \end{array}$$ (22)

where ${\hat{y}}_{i}\in {\mathbb{R}}^{C}$ is the vector of predicted scores or probabilities for the *C* classes for the point/pixel *i*. Applying *S* to all fused features yields ${\hat{y}}_{i},{i=1}^{M}$, which corresponds to the semantic segmentation of the projected point cloud (in image space). Finally, these predictions ${\hat{y}}_{i}$ are mapped back to the original points in *P* (since each point had a correspondence *i* in the fused feature set) to obtain the label predictions ${\hat{\ell }}_{k}$ for each 3D point *p*_*k*_. In summary, the attention-guided fusion ensures that each point’s label decision is informed by both its 3D geometric context and 2D appearance context. This design effectively leverages the complementary nature of the modalities: for example, points on a power line (thin geometry) get support from the image (distinct color against sky), while points on a road (uniform color) get support from 3D structure (flat horizontal surface spanning a large area). The end result is a more accurate and consistent segmentation of the urban scene, with fine details preserved and global context understood.

### 3.5 Loss function

Training RPINet involves a composite loss function that aligns the model to multiple objectives: preserving 3D information through projection, correctly classifying each point, ensuring region-level accuracy, maintaining boundary precision, and enforcing spatial smoothness. We define the total loss as a weighted sum of five terms:


$$\begin{array}{c} L_{\text{total}}=\lambda_{1}L_{\text{info}}+\lambda_{2}L_{\text{CE}}+\lambda_{3}L_{\text{Dice}}+\lambda_{4}L_{\text{IoU}}+\lambda_{5}L_{\text{smooth}}, \end{array}$$ (23)

where $\lambda_{1},\ldots ,\lambda_{5}$ are hyperparameters that balance the contribution of each loss component. We detail each term below:

#### 3.5.1 Information preservation loss (*L*_info_).

Projecting a point cloud to 2D has the risk of losing some of the 3D structural information (due to occlusions or quantization). To mitigate this, we introduce a loss term based on mutual information (MI) between the original point cloud data and the learned 2D representation. Let *X* denote the random variable representing features of the original 3D points (this could include spatial coordinates, intensity, etc.), and *Z* denote the features of the corresponding 2D projection (for instance, the fused feature map or the final logits for those points). We want the 2D representation to retain as much information about the 3D data as possible. The mutual information between *X* and *Z* is defined as:


$$\begin{array}{c} I(X;Z)=\iint p_{X,Z}(x,z)\log\frac{p_{X,Z}(x,z)}{p_{X}(x)p_{Z}(z)}dxdz, \end{array}$$ (24)

which measures how much knowing *X* reduces uncertainty about *Z* (and vice versa). High mutual information means the two representations are strongly linked (the 2D projection encodes a lot of the original 3D data). We cannot directly maximize *I*(*X*;*Z*) easily, but we can add a loss to discourage its decrease. We define


$$\begin{array}{c} L_{\text{info}}=-I(X;Z), \end{array}$$ (25)

the negative mutual information (so minimizing *L*_info_ is equivalent to maximizing *I*(*X*;*Z*)). In practice, *I*(*X*;*Z*) might be estimated via techniques like a variational lower bound or using a mutual information neural estimator, but conceptually this term pushes the network to preserve 3D details in the 2D fused features. By including *L*_info_, we guide RPINet to project the point cloud in a way that important information (like the presence of a small object or the exact shape of a structure) is not lost. This improves segmentation fidelity because the 2D feature map can still differentiate points that were distinct in 3D. For example, if two points are far apart in 3D but happen to project to neighboring pixels, maximizing mutual information will encourage the model to keep their features different enough to reflect the original distance (perhaps via color or slight intensity differences in the projected image), preventing confusion that could degrade segmentation.

#### 3.5.2 Cross-entropy loss (*L*_CE_).

This is the standard supervised segmentation loss that evaluates pixel-wise classification error. After projection and fusion, each point (pixel) *i* has a predicted probability distribution $\hat{y}i$ over the *C* classes (from the softmax output of the segmentation head) and a one-hot ground-truth label vector *y*_*i*_ (where $y_{i,c}=1$ if the true class of point *i* is *c*, otherwise 0). The cross-entropy loss for point *i* is $-\sum_{c=1}^{C}y_{i,c}\log{\hat{y}}_{i,c}$. Averaged (or summed) over all points:


$$\begin{array}{c} L_{\text{CE}}=-\frac{1}{M}\sum_{i=1}^{M}\sum_{c=1}^{C}y_{i,c},\log{\hat{y}}_{i,c}. \end{array}$$ (26)

This term directly optimizes the per-point (pixel) classification accuracy. Minimizing *L*_CE_ encourages the model to assign high probability to the correct class for each point. It provides strong gradient signals especially when the model is very wrong (e.g., if the true class probability is near 0, the loss is large). In our multi-modal setting, cross-entropy alone ensures that each fused feature vector is pushed towards the correct class label. However, cross-entropy treats each point independently and does not account for class imbalance or spatial correlation, which is why we include the following complementary losses.

#### 3.5.3 Dice Loss (*L*_Dice_).

The Dice loss is based on the Sørensen-Dice coefficient, commonly used in segmentation tasks to handle class imbalance and directly maximize overlap between predicted and true regions. For a given class *c*, define the predicted set of points as *P*_*c*_ and the ground truth set as *G*_*c*_. The Dice coefficient for class *c* is:


$$\begin{array}{c} Dice_{c}=\frac{2|P_{c}\cap G_{c}|}{|P_{c}|+|G_{c}|}, \end{array}$$ (27)

which ranges from 0 (no overlap) to 1 (perfect overlap). In terms of the prediction vector ${\hat{y}}_{i}$ and one-hot label *y*_*i*_, an equivalent differentiable form is:


$$\begin{array}{c} Dicec=\frac{2\sum_{i=1}^{M}{\hat{y}}_{i,c},y_{i,c}}{\sum_{i=1}^{M}{\hat{y}}_{i,c}+\sum_{i=1}^{M}y_{i,c}}. \end{array}$$ (28)

We turn this into a loss by averaging over classes and subtracting from 1 (so higher overlap yields lower loss):


$$\begin{array}{cc} L_{\text{Dice}} & =1-\frac{1}{C}\sum_{c=1}^{C}{Dice}_{c}\\ & =1-\frac{2}{C}\sum_{c=1}^{C}\frac{\sum_{i}{\hat{y}}_{i,c},y_{i,c}}{\sum_{i}{\hat{y}}_{i,c}+\sum iy_{i,c}}. \end{array}$$ (29)

Dice loss is particularly useful when classes are imbalanced (e.g., in urban data, “building” might have far more points than “pole” or “tree”). Cross-entropy could be dominated by large classes, whereas Dice loss will force the model to not ignore small classes, since a missing small region dramatically lowers the Dice score for that class. By minimizing *L*_Dice_, RPINet is encouraged to maximize the overlap of its predictions with ground truth for each class, improving the segmentation of both majority and minority classes. In effect, Dice loss adds a region-level focus: it cares about correctly predicting all points of an object (true positives) and not predicting too many that are not there (false positives), complementing cross-entropy’s pointwise focus.

#### 3.5.4 IoU loss (*L*_IoU_).

The Intersection-over-Union (IoU) metric, or Jaccard index, is another overlap-based measure that is harsher than Dice for penalizing discrepancies. For class *c*, ${IoU}_{c}=\frac{\left|P_{c}\cap G_{c}\right|}{\left|P_{c}\cup G_{c}\right|}$. We define the IoU loss as:


$$\begin{array}{cc} L_{\text{IoU}} & =1-\frac{1}{C}\sum_{c=1}^{C}{IoU}_{c}\\ & =1-\frac{1}{C}\sum_{c=1}^{C}\frac{\sum_{i}{\hat{y}}_{i,c},y_{i,c}}{\sum_{i}{\hat{y}}_{i,c}+\sum_{i}y_{i,c}-\sum_{i}{\hat{y}}_{i,c},y_{i,c}}. \end{array}$$ (30)

IoU is similar to Dice but puts more penalty on false positives and false negatives when the regions only partially overlap. By minimizing *L*_IoU_, the model is pushed to achieve high precision and recall for each class segmentation. In particular, this loss sharpens the segmentation boundaries and overall shapes: it is not enough to just cover the ground truth region (as Dice would emphasize), but one must also avoid covering outside the ground truth. In practice, the combination of Dice and IoU losses helps the network output segmentations that are both inclusive of all true pixels and exclusive of wrong pixels. For example, consider a thin object like a lamppost: cross-entropy might struggle if it’s a small fraction of points; Dice will ensure we try to catch it; IoU will further ensure we do not predict it too thick or in slightly the wrong place, as any extra predicted area not in ground truth hurts IoU.

#### 3.5.5 Smoothness regularization (*L*_smooth_).

While the above losses focus on accuracy, we also include a spatial smoothness prior to discourage isolated misclassifications and encourage coherence in the predicted segmentation map (especially in the 2D projection domain). We implement this by penalizing differences in the prediction between neighboring pixels in the 2D projection image. Define a neighborhood *N*_2*D*_(*i*) for each pixel (for instance, the 4-connected or 8-connected neighboring pixels on the grid). We treat $\hat{y}i$ as a vector of class probabilities for pixel *i*. A smoothness loss can be written as:


$$\begin{array}{c} L_{\text{smooth}}=\frac{1}{M}\sum_{i=1}^{M}\sum_{j\in N_{2D}(i)}|{\hat{y}}_{i}-\hat{y}j|2^{2}, \end{array}$$ (31)

where we sum the squared difference between the prediction vectors of adjacent pixels *i* and *j*. This is analogous to a discrete Laplacian or total variation regularizer on the output map, which tries to make neighboring pixels have similar label distributions. The effect is that the model is penalized if it produces a “noisy” segmentation where labels fluctuate rapidly from pixel to pixel without a strong reason. Minimizing *L*_smooth_ encourages more uniform regions of the same class, which aligns with the fact that objects or surfaces in the real world are usually composed of contiguous points. This regularization is careful not to oversmooth real boundaries: at true object edges, the cross-entropy, Dice, and IoU losses already strongly encourage the model to change class, so the smoothness loss will be counteracted by those terms. Essentially, *L*_smooth_ removes salt-and-pepper artifacts (e.g., a single point of building predicted amidst a road region) unless the data truly supports such a change. In the context of RPINet, where predictions are made on the 2D projected image, this loss helps maintain spatial consistency in that image plane which translates back to spatial consistency in 3D as well (neighboring points in the cloud that fall on nearby pixels should have the same label if they belong to the same surface).

Overall, this multi-term loss ensures that RPINet not only achieves high point-wise classification accuracy (through cross-entropy) but also yields segmentations that are information-preserving (through *L*_info_), balanced across classes and overlapping well with ground truth regions (through Dice and IoU losses), and spatially coherent (through the smoothness term). By adjusting the weights $\lambda_{1}\ldots \lambda_{5}$, we can emphasize certain aspects; for example, a higher $\lambda_{1}$ forces the model to be very faithful to 3D structure (useful if projection distortion is a concern), while higher $\lambda_{5}$ will produce cleaner segmentation maps at the potential cost of some boundary precision. In our experiments, we find that incorporating all these terms yields the best overall performance, as each term addresses a different failure mode of large-scale segmentation: *L*_info_ protects against losing 3D details during projection, *L*_CE_ optimizes per-point accuracy, *L*_Dice_ and *L*_IoU_ improve region-level and boundary segmentation quality (especially for small or imbalanced classes), and *L*_smooth_ improves the visual and qualitative coherence of the predictions. Consequently, the trained RPINet model produces segmentation outputs that are not only accurate in labeling each point, but also preserve the integrity of objects and surfaces across the entire urban scene.

## 4 Experiment

### 4.1 Experimental setup

We evaluate our proposed RPINet on the widely used **SensatUrban dataset**, a large-scale, high-resolution, and real-world urban point cloud benchmark that presents unique challenges for semantic segmentation. The dataset contains more than **2.8 billion points** captured by airborne LiDAR over three distinct UK cities: Birmingham, Cambridge, and York. Each city features varied architectural styles, terrain elevation, object densities, and semantic compositions, which makes the dataset particularly suitable for assessing the robustness and generalizability of large-scale segmentation models. The point clouds are annotated with **13 semantic classes**, including roads, buildings, trees, vehicles, and sidewalks, thus covering a broad spectrum of urban elements.

To ensure a standardized evaluation protocol, we adopt the official split of the dataset into 60% for training, 20% for validation, and 20% for testing. All models are trained and evaluated under the same hardware and software environment, specifically using an NVIDIA RTX 3090 GPU with 24GB memory. The implementation is based on PyTorch 1.13, and the training process spans **120 epochs**, with an initial learning rate of **0.001** using the Adam optimizer. A cosine annealing schedule is employed to gradually reduce the learning rate, facilitating stable convergence. The batch size is set to **16**, and we use a hybrid loss function composed of five terms (mutual information, cross-entropy, Dice, IoU, and smoothness regularization), with weights $\lambda_{1}=0.5$, $\lambda_{2}=1.0$, $\lambda_{3}=0.5$, $\lambda_{4}=0.5$, and $\lambda_{5}=0.2$ determined empirically through preliminary validation.

We use three comprehensive and complementary metrics for performance evaluation: 1) **Mean Intersection-over-Union (mIoU)**: the average IoU across all semantic categories, capturing both precision and recall of the segmentation. 2) **Overall Accuracy (OA)**: the ratio of correctly classified points to the total number of points, reflecting the global classification capability. 3) **Mean Class Accuracy (mAcc)**: the average of per-class accuracies, highlighting the model’s performance on less frequent or imbalanced categories. These metrics together provide a holistic evaluation of both per-point and per-class segmentation quality, especially under the high noise, occlusion, and density variations inherent to urban-scale point clouds.

### 4.2 Performance comparison

The overall performance comparison between RPINet and several state-of-the-art methods is presented in Table 1. Our method consistently outperforms all baselines across the three main metrics: mIoU, OA, and mAcc. Notably, RPINet achieves an mIoU of **66.5%**, a clear improvement of **3.7%** over the previous best method, FusionTransformer. Moreover, RPINet achieves the highest OA of **92.3%** and a mean class accuracy of **69.0%**, indicating not only excellent overall performance but also strong ability to handle category imbalance, which is common in urban data (e.g., vehicles or poles occupy much smaller spatial proportions than roads or vegetation).

**Table 1:** Performance comparison on SensatUrban dataset.

Method	mIoU (%)	OA (%)	mAcc (%)	Params (M)	Time (ms)
RandLA-Net [19]	56.1	88.5	59.8	12.4	76
SPVNAS [21]	58.7	89.4	61.3	25.6	85
KPConv [20]	61.2	90.1	63.7	18.9	92
FusionTransformer	62.8	90.8	65.1	31.5	108
**RPINet (Ours)**	**66.5**	**92.3**	**69.0**	**22.1**	**69**

In addition to accuracy, we also report the model size and inference time. RPINet strikes a balance between performance and efficiency, with a moderate parameter count of **22.1M** and an inference time of **69 ms**, making it suitable for large-scale deployment. Despite being lighter than FusionTransformer (31.5M), RPINet achieves significantly better results due to its more effective dual-branch design and hybrid attention mechanism. These results demonstrate the effectiveness of integrating 2D texture features with 3D structural cues via our novel architecture.

### 4.3 Class-wise performance

To better understand the detailed performance of RPINet across different semantic categories, we report the per-class mIoU in Table 2. RPINet achieves the highest scores in all listed classes, outperforming the baselines by a clear margin. Notably, the model performs exceptionally well in **Buildings (77.5%)**, **Roads (73.2%)**, and **Vehicles (68.4%)**, which are particularly challenging due to their sharp boundaries, variable scales, and frequent occlusions.

**Table 2:** Class-wise mIoU comparison on SensatUrban.

Category	RandLA	KPConv	FusionTrans	RPINet
Buildings	68.4	71.2	73.8	**77.5**
Roads	64.1	67.9	69.5	**73.2**
Vehicles	58.9	62.3	63.7	**68.4**
Sidewalks	55.2	58.5	60.1	**63.9**
Vegetation	60.4	63.7	65.2	**68.8**
Misc.	48.9	52.6	54.3	**57.1**

The consistent improvement across categories highlights the advantage of our hybrid fusion approach. 2D remote imagery provides rich texture and color cues that help distinguish semantically similar but geometrically different classes (e.g., vegetation vs. building facades), while the 3D point cloud contributes detailed geometric structure. The Gaussian splatting projection retains fine spatial continuity during 2D conversion, and the multi-scale feature fusion captures both global layout and local geometry. These complementary strengths enable RPINet to produce high-quality, fine-grained, and semantically coherent segmentation maps across the full scene.

### 4.4 Ablation study

We perform a detailed ablation study to assess the individual contributions of each component in RPINet, as summarized in Table 3. Starting from a PointNet++ baseline, we sequentially introduce the Transformer module, Graph Convolutional Networks (GCN), Adaptive Sampling, and the Remote Image (RI) Branch. Each addition results in a measurable improvement in mIoU, validating the effectiveness of the design choices.

**Table 3:** Ablation study of RPINet.

Configuration	mIoU (%)
PointNet++ only	58.9
+ Transformer	60.5
+ GCN	61.4
+ Adaptive Sampling	62.1
+ RI Branch (Gaussian + CNN)	63.0
+ Simple Fusion	64.2
+ **Hybrid Attention Fusion (full)**	**66.5**

The Transformer contributes +1.6% by enabling long-range context modeling, essential for capturing large-scale structures like buildings or roads. The GCN module adds another +0.9% by enhancing local feature consistency and enforcing semantic smoothness across point neighborhoods. Adaptive Sampling improves performance by +0.7%, focusing computational effort on informative regions and reducing redundancy in dense areas.

The introduction of the RI branch and the subsequent fusion of 2D and 3D features contributes a substantial +1.1%. Replacing simple concatenation with our hybrid attention mechanism leads to the largest improvement (+2.3%), clearly demonstrating the superiority of the proposed attention-guided fusion strategy in capturing inter-modal dependencies. These results confirm that each component of RPINet contributes significantly to its final performance.

**Fig 3:**
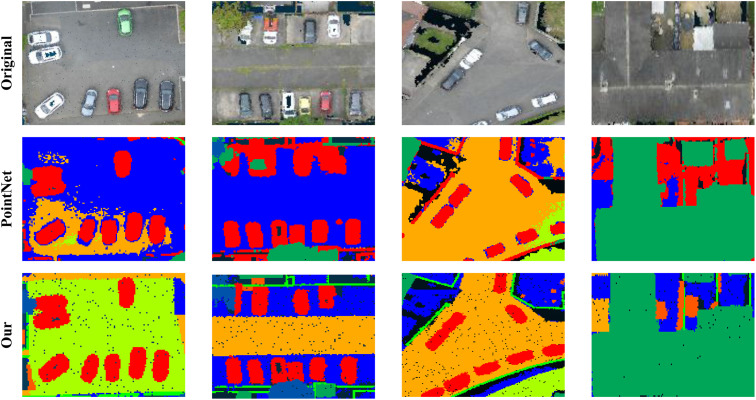
Visual analysis comparing PointNet and our model. Original image patches in this figure are adapted from the SensatUrban dataset by Hu et al. [1] and used under the MIT License from the official SensatUrban repository.

To better understand the effectiveness of our segmentation results, we visualize the qualitative predictions of RPINet in comparison with ground truth and baseline methods. As shown in Fig 3, our method accurately delineates fine object boundaries and preserves semantic consistency across large-scale scenes. The integration of 2D texture cues from remote imagery significantly enhances the delineation of object edges, while the attention-guided fusion allows for consistent predictions in occluded or geometrically ambiguous regions. These visual results confirm that RPINet produces more coherent and detail-preserving segmentation maps compared to existing approaches.

### 4.5 Sampling strategy analysis

Point cloud segmentation on large-scale scenes poses significant computational challenges due to the massive number of points involved. Efficient sampling strategies are critical for improving both performance and speed. Table 4 compares several sampling strategies used in our framework: random sampling, uniform grid sampling, Farthest Point Sampling (FPS), and our proposed adaptive sampling.

**Table 4:** Effect of sampling strategies.

Sampling Method	mIoU (%)	Time (ms)
Random Sampling	63.4	72
Uniform Grid	64.1	80
FPS	64.8	85
**Adaptive Sampling**	**66.5**	**69**

While random and uniform sampling are fast, they fail to preserve semantic diversity and often discard boundary or minority-class points. FPS improves coverage but lacks semantic awareness. Our adaptive sampling method, which incorporates geometric saliency and feature uncertainty, yields the best mIoU (**66.5%**) and maintains competitive inference time (**69 ms**). This confirms that selecting semantically informative and spatially diverse points plays a key role in enhancing segmentation quality.

### 4.6 Generalization on unseen dataset

To evaluate the generalization capability of RPINet, we perform a zero-shot transfer test on the **Toronto-3D dataset** without any fine-tuning. This dataset differs significantly from SensatUrban in terms of sensor characteristics, urban layout, and annotation definitions. As shown in Table 5, RPINet outperforms all compared methods, achieving an mIoU of **62.3%**, which is **3.4%** higher than FusionTransformer.

**Table 5:** Generalization to toronto-3D dataset.

Method	mIoU (%)
RandLA-Net	51.4
SPVNAS	54.7
KPConv	57.2
FusionTransformer	58.9
**RPINet (Ours)**	**62.3**

This result highlights RPINet’s robustness and adaptability to new environments. Its strong generalization is attributed to the fusion of complementary modalities, the use of mutual information loss for information preservation, and multi-scale hierarchical modeling. These design choices allow the model to retain semantic consistency even in unfamiliar domains, making it well-suited for practical deployment in varied urban settings.

## 5 Conclusion

In this paper, we propose **RPINet**, a Remote-Projection and Intelligent Network for large-scale urban point cloud semantic segmentation. RPINet introduces a dual-branch architecture and hybrid attention fusion strategy to effectively integrate 2D remote imagery and 3D point cloud data. Extensive experiments on the SensatUrban and Toronto-3D datasets demonstrate that RPINet achieves state-of-the-art performance in terms of mIoU, OA, and mAcc, while maintaining computational efficiency.

Our ablation study validates the contribution of each module, especially the hybrid attention mechanism and adaptive sampling strategy. RPINet not only improves fine-grained segmentation but also generalizes well across diverse urban scenes. Future work includes exploring real-time inference optimization and extending RPINet to dynamic and multi-temporal scene understanding.

## References

[1] Hu Q, Yang B, Khalid S, Xiao W, Trigoni N, Markham A. Towards Semantic Segmentation of Urban-Scale 3D Point Clouds: A Dataset, Benchmarks and Challenges. In: 2021 IEEE/CVF Conference on Computer Vision and Pattern Recognition (CVPR), 2021. 4975–85. 10.1109/cvpr46437.2021.00494

[2] Fang Z, Hu S, Wang J, Deng Y, Chen X, Fang Y. Prioritized Information Bottleneck Theoretic Framework With Distributed Online Learning for Edge Video Analytics. IEEE Trans Netw. 2025;33(3):1203–19. doi: 10.1109/ton.2025.3526148

[3] Landrieu L, Simonovsky M. Large-Scale Point Cloud Semantic Segmentation with Superpoint Graphs. In: 2018 IEEE/CVF Conference on Computer Vision and Pattern Recognition, 2018. 4558–67. 10.1109/cvpr.2018.00479

[4] Wang L, Huang Y. A Survey of 3D Point Cloud and Deep Learning-Based Approaches for Scene Understanding in Autonomous Driving. IEEE Intell Transport Syst Mag. 2022;14(6):135–54. doi: 10.1109/mits.2021.3109041

[5] Liu P, Ge Y, Duan L, Li W, Luo H, Lv F. Transferring Multi-Modal Domain Knowledge to Uni-Modal Domain for Urban Scene Segmentation. IEEE Trans Intell Transport Syst. 2024;25(9):11576–89. doi: 10.1109/tits.2024.3382880

[6] Bello SA, Yu S, Wang C, Adam JM, Li J. Review: Deep Learning on 3D Point Clouds. Remote Sensing. 2020;12(11):1729. doi: 10.3390/rs12111729

[7] Yan H, Lau A, Fan H. Evaluating Deep Learning Advances for Point Cloud Semantic Segmentation in Urban Environments. KN J Cartogr Geogr Inf. 2025;75(1):3–22. doi: 10.1007/s42489-025-00185-1

[8] Chen J, Kira Z, Cho YK. Deep Learning Approach to Point Cloud Scene Understanding for Automated Scan to 3D Reconstruction. J Comput Civ Eng. 2019;33(4). doi: 10.1061/(asce)cp.1943-5487.0000842

[9] Laupheimer D, Haala N. Multi-modal semantic mesh segmentation in urban scenes. ISPRS Ann Photogramm Remote Sens Spatial Inf Sci. 2022;V-2–2022:267–74. doi: 10.5194/isprs-annals-v-2-2022-267-2022

[10] Huang X, Deng H, Zhang W, Song R, Li Y. Towards Multi-Modal Perception-Based Navigation: A Deep Reinforcement Learning Method. IEEE Robot Autom Lett. 2021;6(3):4986–93. doi: 10.1109/lra.2021.3064461

[11] Guo Y, Wang H, Hu Q, Liu H, Liu L, Bennamoun M. Deep Learning for 3D Point Clouds: A Survey. IEEE Trans Pattern Anal Mach Intell. 2021;43(12):4338–64. doi: 10.1109/TPAMI.2020.3005434 32750799

[12] Lu H, Shi H. Deep learning for 3d point cloud understanding: a survey; 2020. arXiv preprint arXiv. 2009.

[13] Lei Y, Wang Z, Chen F, Wang G, Wang P, Yang Y. Recent advances in multi-modal 3d scene understanding: A comprehensive survey and evaluation. 2023. https://arxiv.org/abs/2310.15676

[14] Bai Y, Fu K. A Large Language Model-based Fake News Detection Framework with RAG Fact-Checking. In: 2024 IEEE International Conference on Big Data (BigData), 2024. 8617–9. 10.1109/bigdata62323.2024.10826000

[15] Pan D, Wu B-N, Sun Y-L, Xu Y-P. A fault-tolerant and energy-efficient design of a network switch based on a quantum-based nano-communication technique. Sustainable Computing: Informatics and Systems. 2023;37:100827. doi: 10.1016/j.suscom.2022.100827

[16] Li H, Chen J, Zheng A, Wu Y, Luo Y. Day-night cross-domain vehicle re-identification. In: Proceedings of the IEEE/CVF Conference on Computer Vision and Pattern Recognition, 2024. 12626–35.

[17] Fang Z, Wang J, Ma Y, Tao Y, Deng Y, Chen X, et al. R-ACP: Real-Time Adaptive Collaborative Perception Leveraging Robust Task-Oriented Communications. IEEE J Sel Areas Commun. 2025;43(12):4215–30. doi: 10.1109/jsac.2025.3623179

[18] Qi CR, Yi L, Su H, Guibas LJ. PointNet: Deep hierarchical feature learning on point sets in a metric space. In: Advances in Neural Information Processing Systems, 2017.

[19] Hu Q, Yang B, Xie L, Rosa S, Guo Y, Wang Z, et al. RandLA-Net: Efficient Semantic Segmentation of Large-Scale Point Clouds. In: 2020 IEEE/CVF Conference on Computer Vision and Pattern Recognition (CVPR), 2020. 11105–14. 10.1109/cvpr42600.2020.01112

[20] Thomas H, Qi CR, Deschaud J-E, Marcotegui B, Goulette F, Guibas L. KPConv: Flexible and Deformable Convolution for Point Clouds. In: 2019 IEEE/CVF International Conference on Computer Vision (ICCV), 2019. 6410–9. 10.1109/iccv.2019.00651

[21] Tang H, Liu Z, Zhao S, Lin Y, Lin J, Wang H, et al. Searching Efficient 3D Architectures with Sparse Point-Voxel Convolution. Lecture Notes in Computer Science. Springer International Publishing. 2020. 685–702. 10.1007/978-3-030-58604-1_41

[22] Qi CR, Su H, NieBner M, Dai A, Yan M, Guibas LJ. Volumetric and Multi-view CNNs for Object Classification on 3D Data. In: 2016 IEEE Conference on Computer Vision and Pattern Recognition (CVPR), 2016. 5648–56. 10.1109/cvpr.2016.609

[23] Li R, Li X, Heng P-A, Fu C-W. PointAugment: An Auto-Augmentation Framework for Point Cloud Classification. In: 2020 IEEE/CVF Conference on Computer Vision and Pattern Recognition (CVPR), 2020. 6377–86. 10.1109/cvpr42600.2020.00641

[24] Zhao H, Jiang L, Jia J, Torr P, Koltun V. Point Transformer. In: 2021 IEEE/CVF International Conference on Computer Vision (ICCV), 2021. 16239–48. 10.1109/iccv48922.2021.01595

[25] Chen X, Liu S, Zhang C, Zhao Y. In: Proceedings of the AAAI Conference on Artificial Intelligence, 2022. 27–35.

